# LCE: an open web portal to explore gene expression and clinical associations in lung cancer

**DOI:** 10.1038/s41388-018-0588-2

**Published:** 2018-12-07

**Authors:** Ling Cai, ShinYi Lin, Luc Girard, Yunyun Zhou, Lin Yang, Bo Ci, Qinbo Zhou, Danni Luo, Bo Yao, Hao Tang, Jeffrey Allen, Kenneth Huffman, Adi Gazdar, John Heymach, Ignacio Wistuba, Guanghua Xiao, John Minna, Yang Xie

**Affiliations:** 1 Quantitative Biomedical Research Center, Department of Clinical Sciences, UT Southwestern Medical Center, 5323 Harry Hines Blvd, Dallas, TX 75390 USA; 2 Children’s Medical Center Research Institute, UT Southwestern Medical Center, 5323 Harry Hines Blvd, Dallas, TX 75390 USA; 3 Bioinformatics Core Facility, UT Southwestern Medical Center, 5323 Harry Hines Blvd, Dallas, TX 75390 USA; 4 Hamon Center for Therapeutic Oncology Research, University of Texas Southwestern Medical Center, 5323 Harry Hines Blvd, Dallas, TX 75390 USA; 5 Department of Data Science, University of Mississippi Medical Center, 2500N State St, Jackson, MS 39216 USA; 6 Department of Pathology, National Cancer Center/Cancer Hospital, Chinese Academy of Medical Sciences and Peking Union Medical College, Beijing, 100021 China; 7 Department of Pathology, University of Texas Southwestern Medical Center, 5323 Harry Hines Blvd, Dallas, TX 75390 USA; 8 Department of Thoracic/Head and Neck Medical Oncology, University of Texas MD Anderson Cancer Center, Houston, TX 77005 USA; 9 Department of Translational Molecular Pathology, University of Texas MD Anderson Cancer Center, Houston, TX 77030 USA; 10 Simmons Cancer Center, University of Texas Southwestern Medical Center, 5323 Harry Hines Blvd, Dallas, TX 75390 USA; 11 Department of Pharmacology, University of Texas Southwestern Medical Center, 5323 Harry Hines Blvd, Dallas, TX 75390 USA; 12 Department of Internal Medicine, University of Texas Southwestern Medical Center, 5323 Harry Hines Blvd, Dallas, TX 75390 USA

**Keywords:** Cancer genomics, Non-small-cell lung cancer

## Abstract

We constructed a lung cancer-specific database housing expression data and clinical data from over 6700 patients in 56 studies. Expression data from 23 genome-wide platforms were carefully processed and quality controlled, whereas clinical data were standardized and rigorously curated. Empowered by this lung cancer database, we created an open access web resource—the Lung Cancer Explorer (LCE), which enables researchers and clinicians to explore these data and perform analyses. Users can perform meta-analyses on LCE to gain a quick overview of the results on tumor vs non-malignant tissue (normal) differential gene expression and expression-survival association. Individual dataset-based survival analysis, comparative analysis, and correlation analysis are also provided with flexible options to allow for customized analyses from the user.

## Introduction

Lung cancer is the leading cause of cancer-related death worldwide. Despite tremendous efforts put toward diagnosis and treatment, the five-year survival rate of lung cancer is still as low as 18% [1]. Over the past few decades, advancements in genome profiling techniques have greatly improved our understanding of cancer development at the molecular level, and have enabled the discovery of biomarkers that facilitate individualized cancer treatments including lung cancer [2]. Recent advances in immune-oncology of lung cancer also show the great importance of marker expression, the tumor mutation burden, and determination of the tumor microenvironment from deposited molecular analyses of lung cancer datasets [3–7]. With the advent of public data repositories of genome profiling data, such as the Gene Expression Omnibus (GEO, [8]), ArrayExpress [9, 10], and The Cancer Genomics Atlas (TCGA), it has become increasingly important and beneficial for researchers to mine the available datasets to discover potential biomarkers and test new biological hypotheses.

Despite the wealth of information offered by such data, utilization of public datasets is not easy, and often it can be prohibitively challenging. There is a plethora of lung cancer patient data published each year, but the data are scattered around in different public data depositories or at individual websites. There are often inconsistencies for the same patient cohort among different websites, likely due to differences in preprocessing approaches and the versions of platform annotations. Moreover, clinical records from different studies are often summarized using different terminologies. Proper usage of publicly available datasets requires specialized expertize in acquiring, processing, normalizing, and filtering of the data, which is challenging for general researchers and clinicians. To facilitate researchers’ use of public datasets for biomarker discovery, a number of re-annotated database have been developed, including OncoMine [11], GeneSapien [12], Gemma [13], M2DB [14], CancerMA [15], cBioPortal [16], KMPlot [17], PrognoScan [18], PROGgene [19] and so forth.

**Fig. 1:**
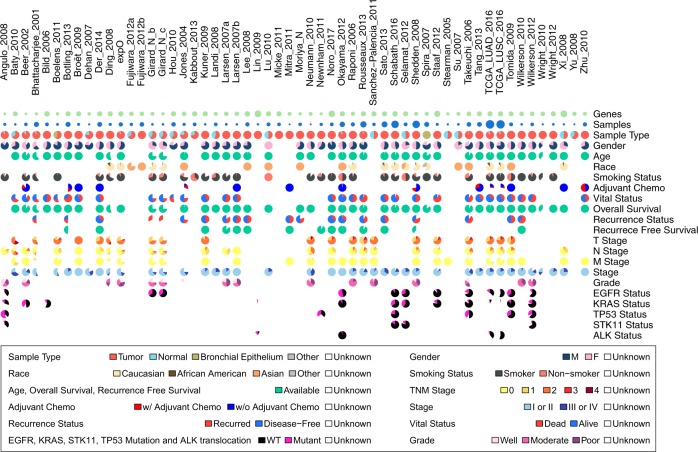
Summary of lung cancer database variable distribution. This summary describes the datasets and features of the lung cancer database that feeds into the Lung Cancer Explorer. Gene expression data and clinical data were collected from 56 studies that include over 6700 patients. For each study and each variable, a pie chart is used to summarize the data. The color scheme for the pie chart sectors are provided below the gridded pie charts. Table S2 provides the specific sample sizes under each category

In this study, we describe our development of a new data commons, Lung Cancer Explorer (LCE) with a web application (http://lce.biohpc.swmed.edu/), populated by a centralized lung cancer database. Compared to other existing databases, our database houses the largest collection of lung tumor expression data from 56 studies for over 6700 patients enriched with rigorously curated clinical data (Fig. 1, Tables S1 and S2). Of special note, tremendous effort was made in manual curation and standardization of the datasets so that they could be used for meta-analysis. This “harmonization” is an important benefit of LCE. Equally important, the user-friendly open web portal provides several easy but versatile analysis tools. These tools include meta-analysis, which enables users to gain a quick overview of the results from all datasets while combining statistical power from multiple datasets, as well as individual dataset-based analyses that allow for more flexibility and customization from the user.

## Results

### Construction of the lung cancer database

Over a span of 5 years, we have collected 56 datasets generated by 23 genome-wide expression platforms (see “Data collection”, “Clinical data curation”, and “Expression data processing” sections in Supplementary Methods). The overarching goal is to include datasets with large numbers of samples, as well as datasets with more comprehensive coverage of clinical information with an emphasis on survival data. The number of samples in the studies we have collected has a median of 100, maximum of 576, and minimum of 27.

**Fig. 2:**
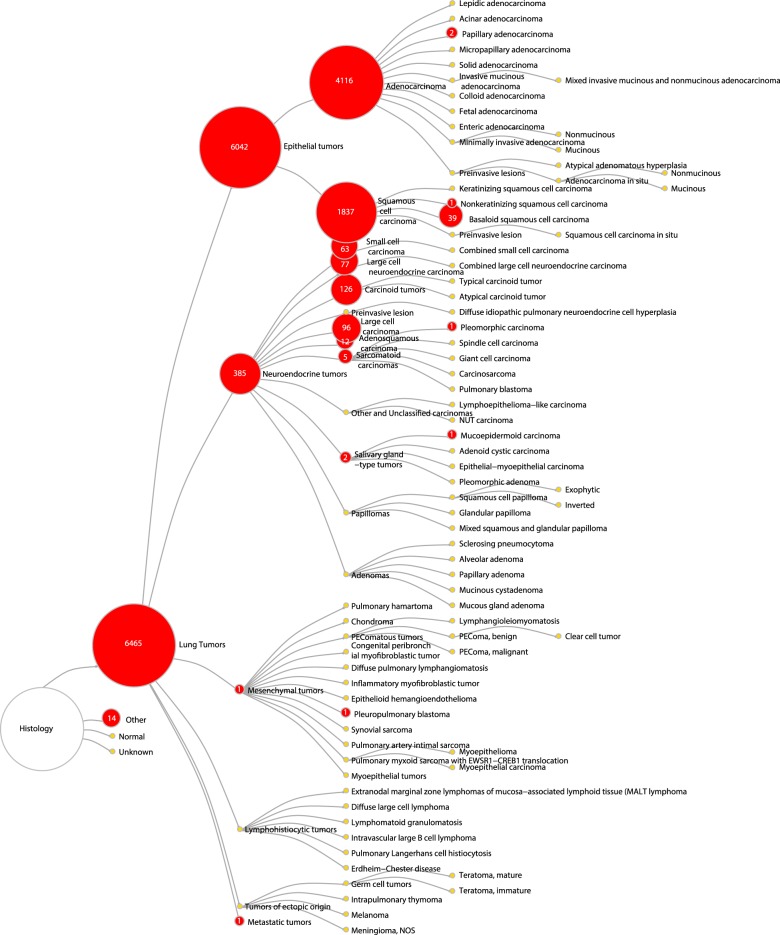
Histology classification of samples collected in the lung cancer database. This tree diagram represents the hierarchical structure of the 2015 WHO classification system of lung tumors. Numbers on the red nodes denote the number of samples from the lung cancer database belonging to the corresponding histology type

The availability and distribution of clinical variables across all studies are summarized in Fig. 1 and Table S2. The clinical variables we collected include tumor histology as defined by the 2015 WHO lung tumor classification system (Fig. 2), as well as patient demographics, diagnosis, adjuvant therapy status, smoking status, recurrence-free and overall survival time and status, and mutation status of some key cancer genes (Fig. 1 and Table S2). Extensive quality control measures were taken for assessment of the expression data and clinical data. Details of these measures are described in the Supplementary Methods (also see Figure S3).

### Lung Cancer Explorer

Having established a high-quality lung cancer database, we constructed the user-friendly website LCE (http://lce.biohpc.swmed.edu), allowing the cancer research community to gain easy access to our resources. Our dataset inventory and sources are described on the DATA page of LCE. Processed data are available for user download under each study. The ANALYSIS page of LCE provides survival analysis, comparative analysis and co-expression analysis tools based on individual datasets, as well as meta-analysis tools based on multiple datasets. The functionality of these tools is described in detail in the following sections.

### Survival analysis in LCE

#### Flexible group dichotomization

Survival analysis is commonly provided in online cancer databases to allow users to assess the association between gene expression and prognosis, and the median is routinely used as the dichotomization cutoff for the continuous gene expression. However, gene expression pattern is often a result of heterogeneous oncogenotypes and the distribution is often unbalanced. The LCE survival analysis module offers four options for cutoff value, including “median”, “mean”, “cluster”, and “custom”. In the Results panel, a Kaplan–Meier plot, table of summary statistics and Kernel density plot of the expression data are provided to the user. The density plot visualizes the distribution of the gene expression and facilitates the user in determining whether they should modify their choice of cutoff. In particular, the “cluster” option in cutoff selection would be a more rational choice for bimodally distributed expression values, as it separates the sample groups by a cutoff estimated from Gaussian mixture modeling.

#### Survival analysis examples with cluster-based cutoff

**Fig. 3:**
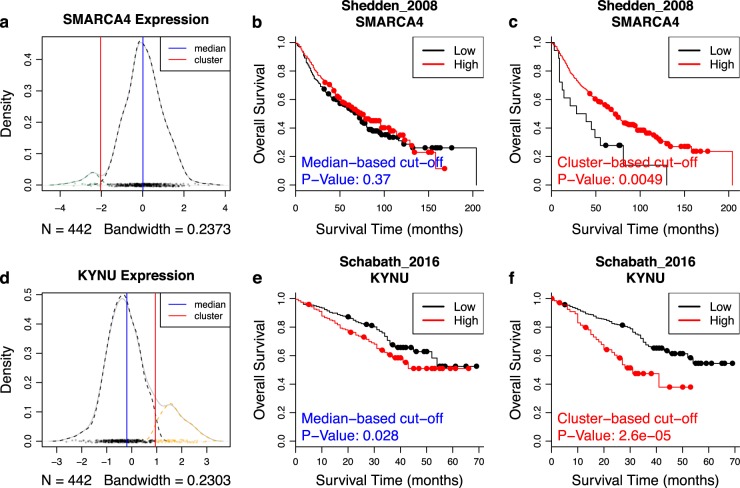
Examples of survival analysis with more significant results when cluster-based cutoff is used. **a** Bi-modal distribution of expression in Shedden_2008 dataset. The solid blue line marks the cutoff at the median, whereas the solid red line marks the cutoff determined by Gaussian mixture model. **b** Kaplan–Meier curves from the survival analysis of Shedden_2008 using groups defined by *SMARCA4* gene expression with cutoff at median. *P*-value from the log-rank test is denoted at the bottom left corner of the plot. **c** Survival analysis of Shedden_2008 using groups defined by Gaussian mixture model of *SMARCA4* expression. **d** Bi-modal distribution of *KYNU* expression in Schabath_2016 dataset. **e** Survival analysis of Schabath_2016 using groups defined by *SMARCA4* gene expression with cutoff at median. **f** Survival analysis of Schabath_2016 using groups defined by Gaussian mixture model of *KYNU* expression

In Fig. 3 we provide examples using the genes *SMARCA4* and *KYNU* in two lung adenocarcinoma (ADC) studies. Bi-modal distribution of gene expression was observed in both cases (Fig. 3a, d). *SMARCA4*, a well-known tumor suppressor gene [20] that can serve as prognostic indicators in non-small cell lung cancer [21] and breast cancer [22], was under-expressed in a small fraction of samples from the Shedden_2008 study [23], and the corresponding patients had worse survival outcome (Fig. 3c). In contrast, *KYNU* was over-expressed in a small proportion of samples in dataset Schabath_2016 [24] and the corresponding patients also had worse survival outcome. In both cases, results from survival analysis were more significant when the cutoff was selected by “cluster” as opposed to “median” (Fig. 3b, c, e, f). With the built-in “cluster” option for cutoff selection, users can easily generate figures like Fig. 3c, f and compare them with the default “median” options like Fig. 3b, e.

#### Analysis stratification by additional clinical variables

**Fig. 4:**
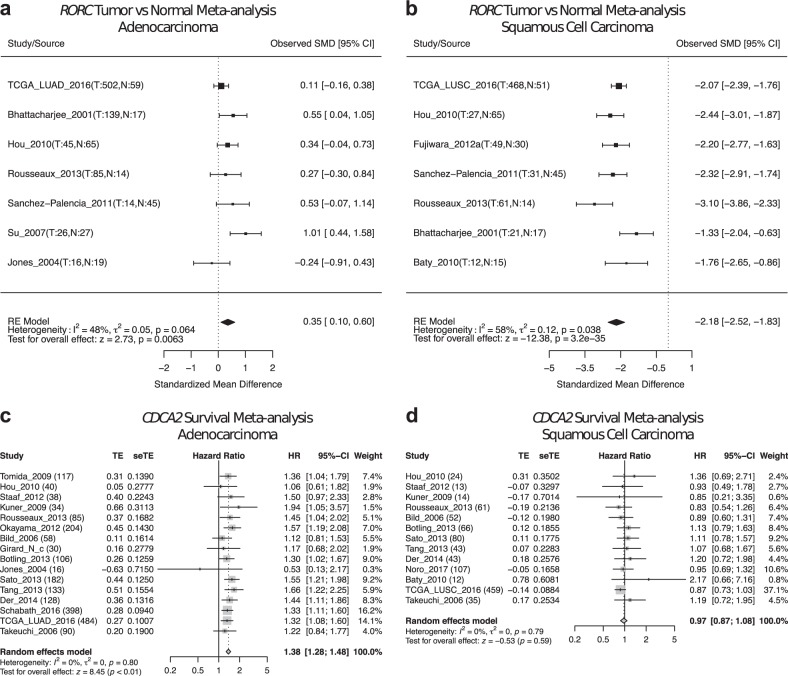
High klotho expression has more significant association with positive survival outcome in males. For each of the six selected studies, survival analysis assessing prognosis association of KL gene expression was performed for male patients or female patients only. In each analysis, the median was used as a cutoff for dichotomizing patients. In all six studies, a more significant association with better prognosis was found in the male patients compared to the female patients

In the LCE survival analysis module, options are provided for users to select a group of patients by age, race, gender, smoking status, and histology. This allows users to assess the association between the expression of a user-selected gene and patient survival (gene-survival association) within a user-defined subpopulation of patients. An example in Fig. 4 is provided to illustrate the advantage of this approach in the identification of a gender-specific gene-survival association. In Fig. 4, association of KL gene expression and survival was tested separately in female and in male patients. We show that for several studies, a stronger positive association between high klotho gene expression and overall survival could be observed in male patients as compared to female patients. Klotho, encoded by gene *KL*, is a well characterized anti-aging gene [25]. It has been observed that the extension of lifespan by klotho overexpression is more pronounced in males than in females [26], and only male but not female klotho mutant mice responded to a phosphorus restriction diet to extend lifespan [27]. In recent years, klotho has also been characterized as a tumor suppressor gene [28]. From our analyses, it is interesting to see that the tumor suppressing effect of klotho also seems to be higher in males than in females (Fig. 4).

### Meta-analysis in LCE

#### Types of meta-analysis

In LCE, meta-analysis tools are provided to allow users to address two questions: (1) differential expression between tumor and normal samples; and (2) survival association of gene expression.

#### Cohort-specific meta-analysis and examples

Results from both types of meta-analyses are visualized as forest plots. We provide three options, “All Cancers”, “Adenocarcinoma” (ADC), or “Squamous Cell Carcinoma” (SCC), to allow users to choose the lung cancer subtype(s) they want to include in the meta-analysis since the survival association and expression difference between tumor and normal could be cancer-type specific.

**Fig. 5:**
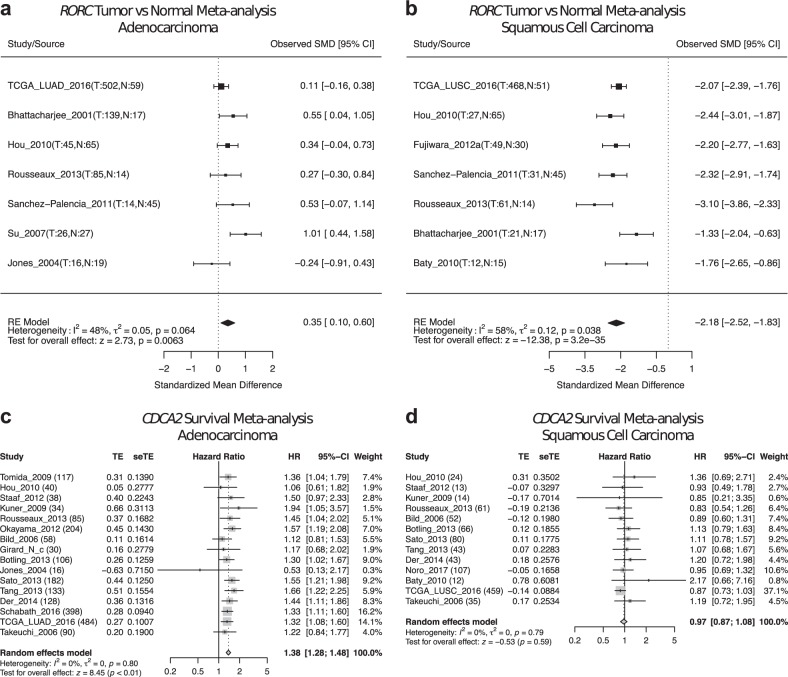
Examples of different meta-analysis results in lung adenocarcinoma vs squamous cell carcinoma. **a**, **b** *RORC* tumor vs normal meta-analyses in lung ADC studies (**a**) and lung SCC studies (**b**). **c**, **d** *CDCA2* survival meta-analyses in lung ADC studies (**a**) and lung SCC studies. Note that differential gene expression meta-analysis for *RORC* is only significant in lung SCC patients, whereas survival meta-analysis for *CDCA2* is only significant in lung ADC patients. In each forest plot, the name of each study is followed by the number of tumor and normal samples (tumor vs normal meta-analysis) or total tumor samples (survival meta-analysis). SMD standardized mean difference, TE estimated treatment effect, seTE standard error of treatment effect, HR hazard ratio, CI confidence interval

For example, with lung cancer subtype-specific meta-analysis, we found consistent downregulation of RAR related orphan receptor C (*RORC*) in multiple lung SCC studies (Fig. 5b) but not in lung ADC studies (Fig. 5a). Interestingly, *RORC* was also previously found in a 3-gene signature to distinguish lung ADC and lung SCC [29]. We also found that in multiple lung ADC studies, expression of cell division cycle-associated protein 2 (*CDCA2*) was associated with worse overall survival outcome (Fig. 5c), whereas this trend was not observed for lung SCC datasets (Fig. 5d).

#### Validation of tumor versus normal gene expression difference meta-analysis

**Fig. 6:**
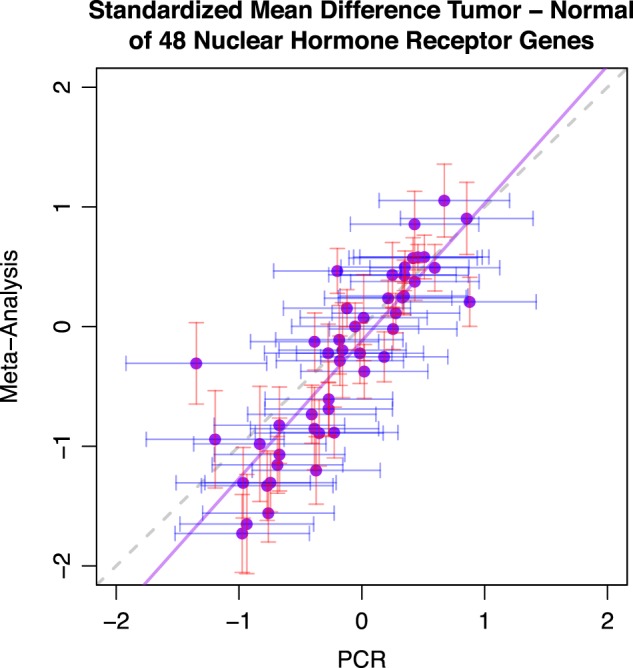
Meta-analysis estimates agree with qPCR measurements on tumor vs normal expression differences for 46 nuclear hormone receptor genes. Results from qPCR measurements of 30 tumor-normal pairs (*x*-axis values) and meta-analysis estimates from 21 studies (*y*-axis values) on gene expression differences between tumor and normal tissues for 46 nuclear hormone receptor genes were used to evaluate consistency between the two approaches. The values on the *x*-axis and *y*-axis are the standardized mean difference estimated by Hedges’ G method. The solid purple line represents a linear regression line, whereas the dashed gray line identifies where *x* equals *y*

With access to qPCR measurements of 46 nuclear hormone receptor genes in 30 pairs of matched tumor and normal lung cancer samples, we were able to compare the standardized mean difference between tumor and normal tissue gene expression estimated from meta-analysis to the qPCR measurement results. A strong agreement was observed between the two results, supporting the validity of our meta-analysis and the high quality of our datasets (Fig. 6, Table S5).

#### Assessing reproducibility across different studies from meta-analysis

**Fig. 7:**
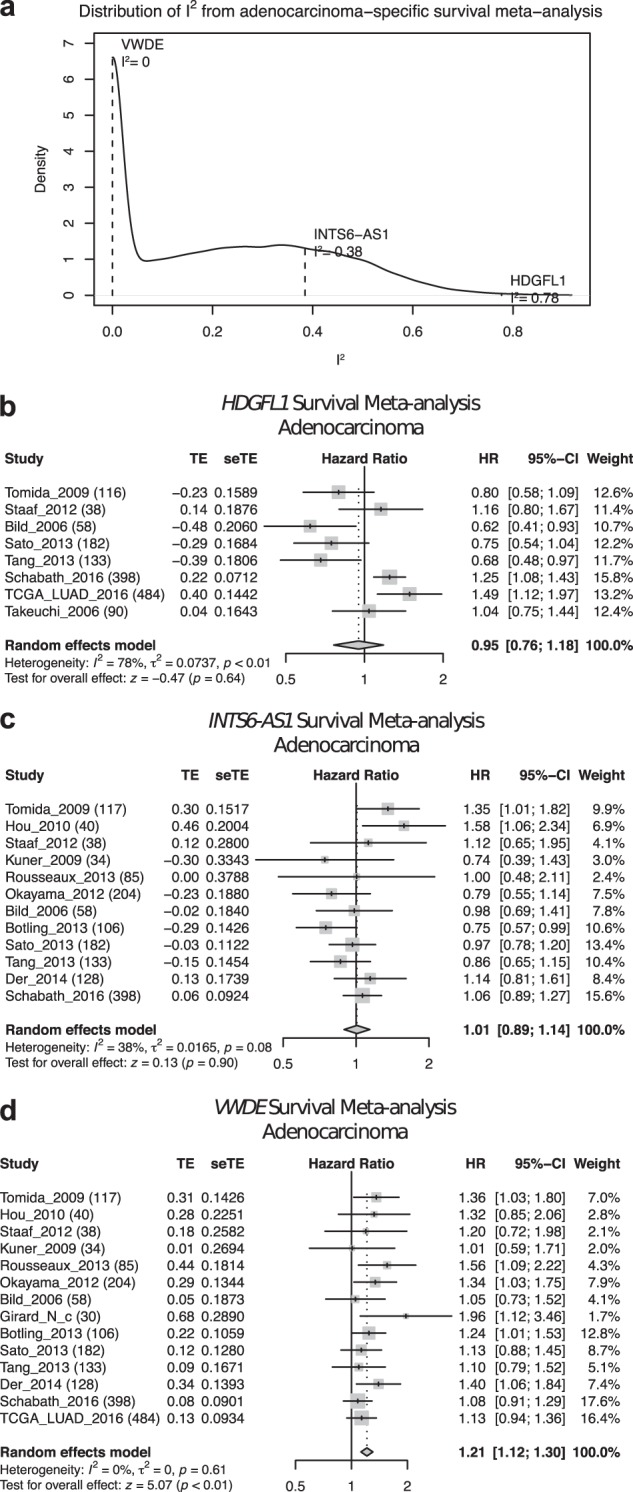
Assessment of result consistency by I^2^ statistics in meta-analysis of survival-gene expression association. **a** Density estimation of I^2^ distribution. Three genes with different I^2^ statistics were selected as examples in (**b**), (**c**), and (**d**). A larger I^2^ value suggests a larger degree of heterogeneity across studies, whereas a smaller I^2^ value is reflective of a higher degree of consistency among studies. **b**, **c**, **d** Example forest plots of survival meta-analysis with different heterogeneity: large (**b**), intermediate (**c**), and small (**d**)

Meta-analysis is a unique tool provided by LCE, as it not only provides users with statistical estimates that are more precise than using any single dataset, it also allows users to recognize the extent of reproducibility of a specific analysis across different datasets. In the forest plots generated by the LCE meta-analysis module, we provide users with a heterogeneity test using the I^2^ statistic, which describes the percentage of variation across studies that is due to heterogeneity [30]. It is important to note that inconsistency in the results between different studies could arise from differences in patient population or sample procurement, as well as in data acquisition. In some cases, the results are more consistent for specific genes than others (Fig. 7). Hence, the meta-analysis tool provided by LCE allows users to identify discrepancies among different datasets in order to estimate the generalizability of the results.

### Comparative analysis in LCE

**Fig. 8:**
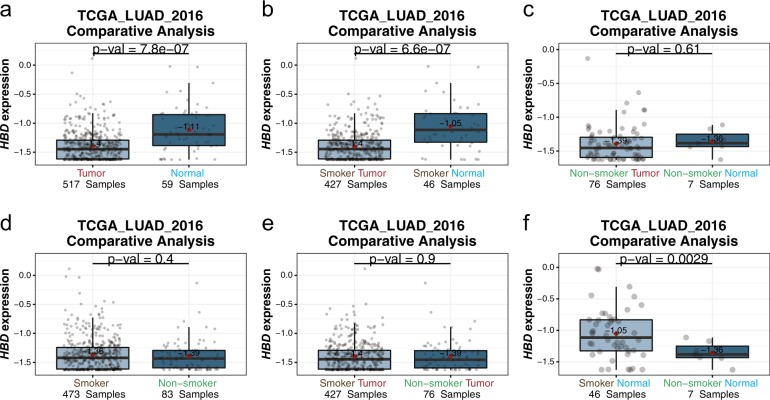
Interaction between sample tissue type and smoking status in HBD gene expression. **a**, **d** Boxplots comparing *HBD* gene expression between two groups dichotomized on a single clinical variable: tissue type (**a**) or smoking status (**d**). **b**, **c**, **e**, **f** Boxplots comparing *HBD* gene expression between two groups defined by a combination of two clinical variables: different tissues in smoker (**b**), different tissues in non-smoker (**c**), tumor from patients with different smoking status (**e**), and normal tissues from patients with different smoking status (**f**)

Comparative analysis was implemented for users to assess the associations between a user-selected gene and clinical factors such as gender, age, histology types, disease stages, etc., within a specific dataset. The expression levels of the selected gene in the user-defined patient groups are shown in boxplots and *p* values of the expression differences are reported. In addition to group assignment based on a single clinical variable, a unique functionality of LCE is that users can define patient groups based on a combination of clinical factors. This provides a great extent of flexibility in hypothesis testing to understand the interactions between different clinical variables. For example, expression comparison of the hemoglobin subunit delta encoding gene *HBD* in the TCGA_LUAD_2016 cohort shows that tumor samples have decreased *HBD* expression compared to normal samples (Fig. 8a), whereas samples from smokers and non-smokers have similar expression levels (Fig. 8d). However, by stratifying patient groups with two factors, both tissue type (tumor vs normal) and smoking status, we find the difference in *HBD* levels between normal and tumor tissues is significant only in smokers but not in non-smokers (Fig. 8b, c), and normal samples from smokers have elevated *HBD* expression compared to normal samples from non-smokers (Fig. 8f). In contrast, no difference in *HBD* expression was observed for tumor tissues from smokers vs non-smokers (Fig. 8b), nor do *HBD* expression levels differ in the tumor and normal tissues of non-smokers (Fig. 8f).

The results from these comparisons suggest that *HBD* expression is upregulated in normal lung tissue by smoking but is downregulated again when tumors form in smokers. We also observed similar trends in the other hemoglobin subunit encoding genes *HBG1*, *HBG2*, and *HBM*, which is consistent with the previous finding that hemoglobin levels increase in smokers [31].

### Correlation analysis in LCE

**Fig. 9:**
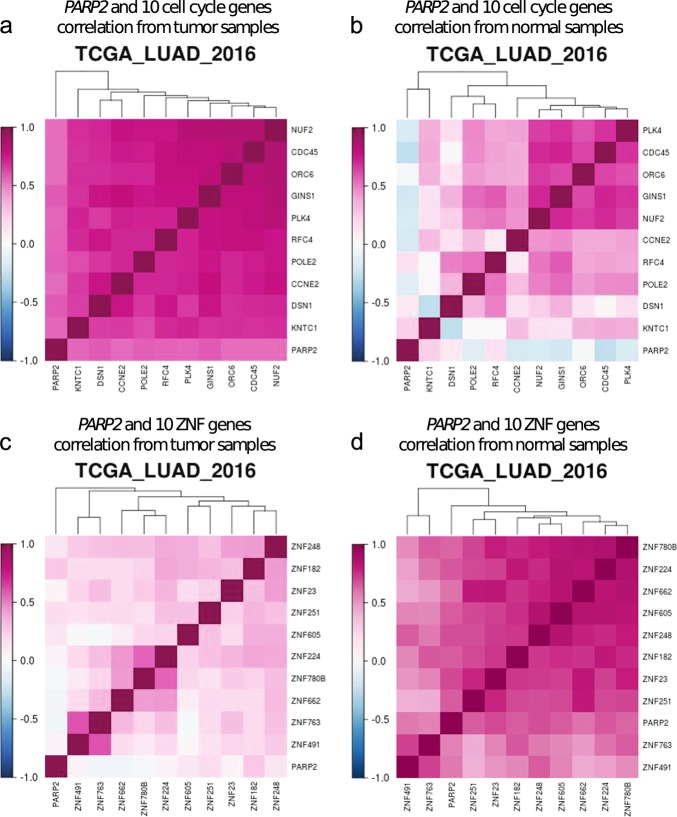
Different co-expression pattern between *PARP2* and cycle genes. **a**, **b**, **c**, **d** Heatmaps of gene–gene correlation matrices from TCGA_LUAD_2016 for *PARP2* and 10 selected cell cycle genes from tumor sample expression data (**a**) or normal sample expression data (**b**), and for *PARP2* and 10 selected C2H2-type zinc finger genes (ZNF) from tumor sample expression data (**c**) or normal sample expression data (**d**). The highly positive correlation between *PARP2* and cell cycle genes was seen only in tumor samples but not normal samples (**a**, **b**), whereas the high degree of positive correlation between *PARP2* and ZNF genes was observed only in normal tissue samples but not tumor samples (**c**, **d**)

The correlation analysis tool from LCE provides users a heatmap to visualize the expression correlations among a list of user-defined genes in user-selected datasets. A high degree of expression correlation of genes often implies functional association, as genes involved in the same pathway or biological function are often subject to concerted regulation at transcription level [32]. Functional partners of the same gene could differ in a tissue-specific manner [33], and the gene network could also re-wire under a different disease context. In LCE we provide three options, “All”, “Lung Tumor” and “Normal”, to allow users to calculate a gene expression correlation matrix based on a specific sample type and subsequently generate a clustered heatmap, which conveniently allows users to identify changes in the co-expression patterns of the user-defined gene list. One such example is provided in Fig. 9, where we show that in tumor, there is a high degree of co-expression between poly(ADP-ribose) polymerase-2 (*PARP2*) and 10 cell cycle genes (Fig. 9a) selected from MSigDB “REACTOME_CELL_CYCLE” gene set [34, 35], whereas this co-expression is diminished in normal tissues (Fig. 9b). This is consistent with the role of *PARP2* in DNA repair [36]; since genomic instability and mutation is a hallmark of cancer, the cancer-specific co-expression of *PARP2* and cell cycle genes may indicate that *PARP2* is actively engaged in DNA repair while cancer cells divide. On the other hand, we found *PARP2* highly correlated with zinc fingers C2H2-type genes (ZNF) [37] in normal but not cancer tissue (Fig. 9c, d). This normal-specific co-expression of *PARP2* and ZNF genes may suggest alternative roles of *PARP2* in transcriptional regulation independent of its DNA repair function.

## Discussion

In this paper, we described the construction of the LCE database for lung cancer gene expression analysis. It was carefully designed for lung cancer researchers to interrogate gene expression association with patient clinical features. As the collected datasets are highly heterogeneous, extensive efforts were put forth to reprocess and normalize expression data from 23 different expression profiling platforms, and a large amount of manual curation work was performed to standardize clinical terminology. Such manual inspection, though time consuming, greatly improves the data accuracy and usability, which sets our work apart from other databases. The resulting database with high-quality datasets enables versatile analysis tools in our LCE. We provide meta-analysis tools that summarize results across multiple datasets in the form of forest plots to allow users to gain a summary view of the overall trend and heterogeneity among studies. We also provide individual dataset-based analysis tools to allow users the flexibility to intricately formulate their analysis to best fit the research question. Results and biological insights we obtained from examples (Figs. 3–5 and 7–9) demonstrated the unique advantages of our tools over the current publically available web tools, as none of these results could have been produced with the existing public tools.

We welcome users to contribute or suggest additional datasets to be evaluated and added to our lung cancer database. Suggestions can be made by leaving a comment at the contact page of LCE. It is in our plan to add a functionality to LCE to enable users to upload their own data to our database and perform analysis with our web application. In the future, we would also like to expand the lung cancer database to include cell line data and patient-derived xenograft (PDX) data. Besides gene expression data, other types of molecular profiling data (such as proteomic data, mutation data, copy number variation data, epigenomics data, microRNA data, etc.) and imaging data (such as H&E pathological slide images) will also be added to the lung cancer database. Separate data tables and supporting data dictionaries will be created for the new molecular data types. We will first identify studies within our collection that possess such data and add them to our database, then look for additional datasets that contain such molecular data as well as clinical data to add to our database. We will also expand the analysis tool repertoire on LCE to include multivariate analysis and other integrative analytical approaches.

Finally, we will conduct a variety of systematic analyses with the lung cancer database to generate testable hypotheses (for example, identification of genes associated with different oncogenotypes, gender, smoking status, etc. followed by gene set enrichment analysis). Results from such systematic analyses will be provided to the lung cancer research community to provoke hypothesis generation, testing, and validation.

## Material and methods

### Data collection and processing

#### Dataset selection

Datasets were collected from GEO, TCGA, and individual literatures. The search of GEO was performed by GEOmetadb [38]. For datasets that had not been deposited into GEO, we made our selection through a literature search and by referencing other commonly used databases.

#### Clinical data curation

Clinical data for datasets deposited into GEO were retrieved from GEO by R package GEOquery; TCGA clinical data were downloaded from Sage Bionetworks’ Synapse database [39], and other datasets were downloaded from sources provided in the original publication. The clinical data obtained directly from these public domains often contained non-standard terminology. To standardize the clinical variables from different studies, codebooks were devised for each variable in order to ensure the accuracy and compatibility of the clinical annotation from different sources (Table S4.1–S4.6). The patient histology codebook was created based on the 2015 World Health Organization (WHO) Classification of Lung Tumors [40] (Fig. 2 and Table S3). In order to facilitate users in integrating our datasets with other cancer datasets, we also provided the ICDO code [40] and the corresponding SNOMED-CT code [41] for histological subtypes in the processed data included on LCE for download. For the TCGA lung cancer data in particular, instead of using the histology classification provided by the patient information file, histology was determined based on expression signature as developed by Girard et al. [42], as that study has shown improved classification accuracy with the gene expression classifier on the TCGA data. Consequently, the histology-misclassified samples were excluded from the TCGA cohorts in cancer-specific meta-analysis. For all datasets, programmatic and manual data curations were carried out and the procedures were repeated three times with scrutiny. For our records, all the data handling steps were saved with detailed documentation.

#### Quality control of clinical data

Manual data curation was performed to ensure consistency between supplementary information associated with the original publication and the clinical data downloaded from GEO. Clinical information found only in the original publication but not in the GEO records was also extracted. Here we describe a few examples of our manual curation from numerous instances: we checked if there were exclusion criteria in the paper that imposed restrictions on adjuvant therapy, tumor stage, etc.; when calculating the survival time we looked for surgical date, and if it was available we used it as the start date for survival time instead of the initial diagnosis date, since the gene expression data reflected the tumor profile on the surgical date; when certain samples were considered low quality and removed from analyses in the associated publication, we followed the same discretion to excl ude such samples from our collection; we removed cell line samples to ensure our collection included exclusively patient samples; when tumor percentage information was available, we removed samples with < 50% tumor content.

#### Expression data processing

Expression data for datasets deposited into GEO were retrieved from GEO by R package GEOquery. TCGA expression data were downloaded from Broad GDAD firehose [43], and other datasets were downloaded from the sources provided in the original research papers. It is not uncommon in the field of biomarker discovery for signatures to have poor reproducibility in other datasets. Such discrepancy could be at least partially attributed to the differences in experimental settings, sample handling, measurement platforms and, importantly, data processing procedures. The datasets collected in this study were generated from 23 different platforms, with the majority being microarrays. We adopted different strategies to process the data (Figure S1) to convert the expression data from probe level to gene level.

#### Quality control of gene expression data

To perform quality control of the expression data input for meta-analysis, a method that checks for reproducibility across studies based on the concept of the integrative correlation coefficient (ICC) [44, 45] was implemented. The premise of this approach is that most of the pairwise gene–gene correlation should be preserved across different studies. The relationship of reproducibility between studies could be visualized by ICC-based clustering, as shown in Figure S3. Considering that some gene–gene correlation could be tissue-type specific, samples of different tissue types from the same study were separated into distinct groups before we calculated the ICC. As expected, in clusters defined by ICC, subgroups of different sample types from the same study in many cases did not cluster together; instead, samples of the same tissue type from different studies tended to cluster together. A clade of four studies with very little correlation with other sample groups was identified. These four studies were removed from subsequent meta-analyses. However, they were still available to use in the individual dataset-based analysis. Moreover, the two RNA-seq datasets from TCGA revealed high correlation with datasets from microarray platforms, supporting the compatibility of datasets from different platforms based on our processing approach.

#### Database structure/web interface

Our web application LCE can be accessed through http://lce.biohpc.swmed.edu/. It was created using PHP (7.0.12-1) in the R Programming environment (3.3.1) with MySQL database (Ver 14.14 Distrib 5.5.49) in the backend. Our MySQL database contains tables for samples, patients and gene expression data with supporting data dictionaries (Figure S2 and Table S4.1–S4.6).

#### Code availability

Data cleaning, processing, and analyses were performed using R. R scripts are available upon request.

### Statistical analysis methods

#### Cluster-based cutoff for patient grouping

In many cases, gene expression follows a bi-modal distribution with unbalanced sample sizes in each group. A cluster-based cutoff selection is provided to assist identification of an optimal cutoff value for group dichotomization in survival analysis. R package *mclust* [46] was used to identify the gene expression cutoff based on Gaussian mixture model clustering, assuming a bi-modal distribution when users select the “cluster” option under the survival analysis module of LCE.

#### Survival analysis

Survival curves were estimated using the product-limit method of Kaplan–Meier [47] (*survival*, R package [48]). A log-rank test was used to compare the survival differences among different patient groups. A Cox proportional hazard regression model was used to assess the survival association and calculate the hazard ratio (HR) with continuous gene expression in each individual dataset.

#### Meta-analysis

For survival meta-analysis, the R package *meta* [49] was used to calculate the summary HR from the HRs of individual datasets. For tumor vs normal differential expression meta-analysis, R package *metafor* [50] was used to calculate the summary standardized mean difference (tumor – normal) using Hedges’ G as an effect size metric.

#### Comparative analysis

For comparative analysis, Welch’s two-sample t-test assuming unequal variance was used to generate the *p*-value. In the resulting box whisker plot, the lower whisker extends from the lower quartile to the lowest smaller value within 1.5 inter-quartile-range (IQR), whereas the upper whisker extends from the upper quartile to the highest larger value within 1.5 IQR. The red solid dot and the value beside it represent the group mean.

#### Correlation analysis

For correlation analysis, the Pearson correlation was used to calculate the correlation coefficients. The dendrogram for the heatmap was generated based on complete-linkage hierarchical clustering of the correlation coefficients based on Euclidean distance.

## Electronic supplementary material

Supplementary Figures

Table S1

Table S2

Table S3

Table S4.1

Table S4.2

Table S4.3

Table S4.4

Table S4.5

Table S4.6

Table S5

## Data Availability

All the datasets were downloaded from the public domain. The processed and normalized data are available upon request. The web-portal we developed in this study can be accessed through the following link: http://lce.biohpc.swmed.edu/
