# Supplementary material for: LCE: an open web portal to explore gene expression and clinical associations in lung cancer
Source: Oncogene. 2018 Dec 7;38(14):2551–64. doi: 10.1038/s41388-018-0588-2 (PMC6477796; doi:10.1038/s41388-018-0588-2)
Supplement: Supplementary file 1 — Supplementary Figures [file 41388_2018_588_MOESM1_ESM.docx]

**
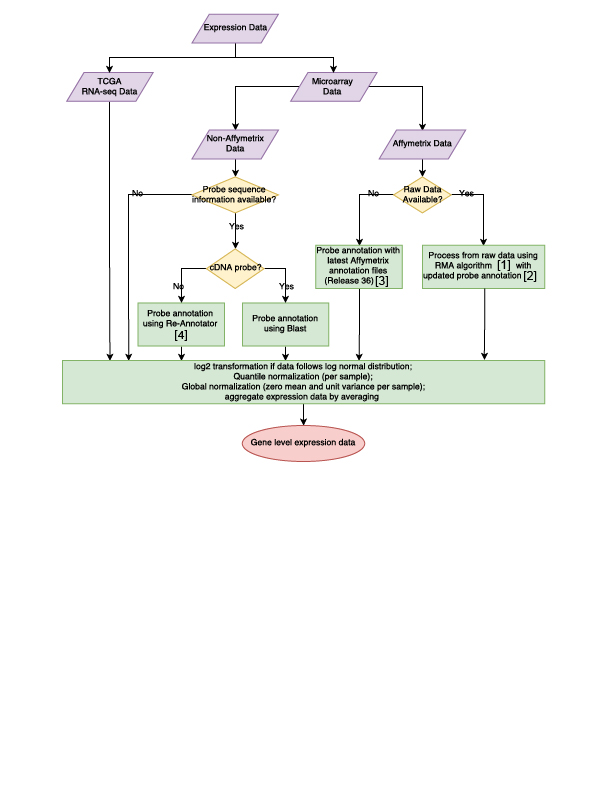
**

**FigS1. Flowchart of expression data processing strategies.**

Different strategies were adopted to process expression data from different platforms with or without raw data available for download. We re-annotated the probes whenever possible because the original probe design and annotations were often based on obsolete transcriptome databases. If the data were generated on an Affymetrix platform with raw data available for download, we processed from the raw data using an RMA algorithm [1] with updated probe annotation [2]; for data generated from the Affymetrix platform without raw data available for download, we mapped the probes to genes based on the most up-to-date probe annotation files (Release 36) downloaded from the Affymetrix website [3]. For data generated on a non-Affymetrix platform with probe sequence information available, re-annotation of datasets with short probes was performed using Re-Annotator [4], whereas datasets with long probes such as cDNA probes had probes remapped by Blast, similar to the strategy we adopted in ProbeMapper [5]. For data without probe sequence information available, we used the vender-provided probe annotation. Once the expression data have been mapped to gene level, we perform normalization steps for all datasets as follows: data was transformed if it followed a log normal distribution, quantile normalization and global normalization were performed subsequently, and the expression data was aggregated so that each sample has a unique expression value per gene.


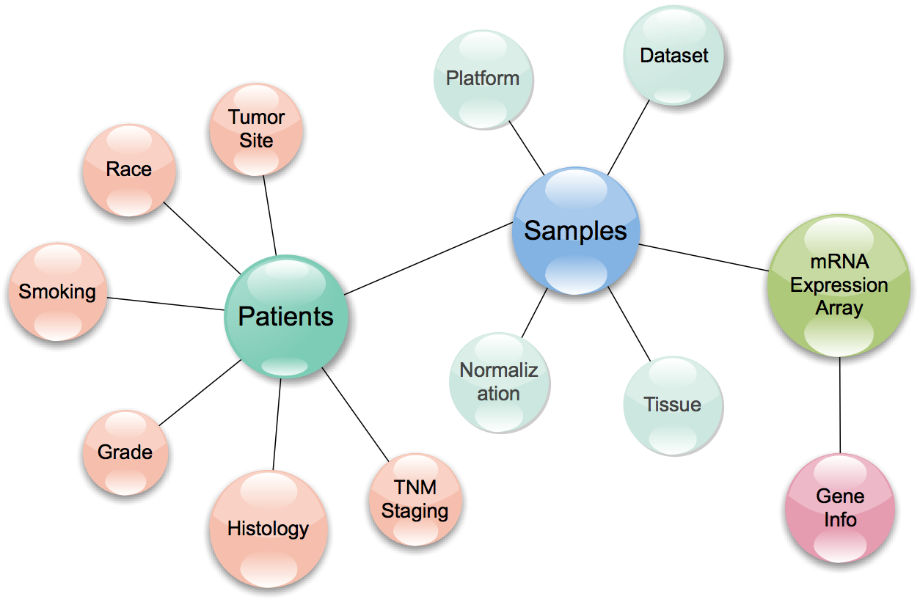


**FigS2. Structure of the lung cancer database.**

This schematic diagram shows the design of main data storage tables surrounded by supporting data dictionaries comprising our relational lung cancer database. Three main data storage tables are the patients table, samples table and the mRNA expression table. The patients table and the samples table are connected by patient ID, whereas the samples table and the mRNA expression table are connected by sample ID.


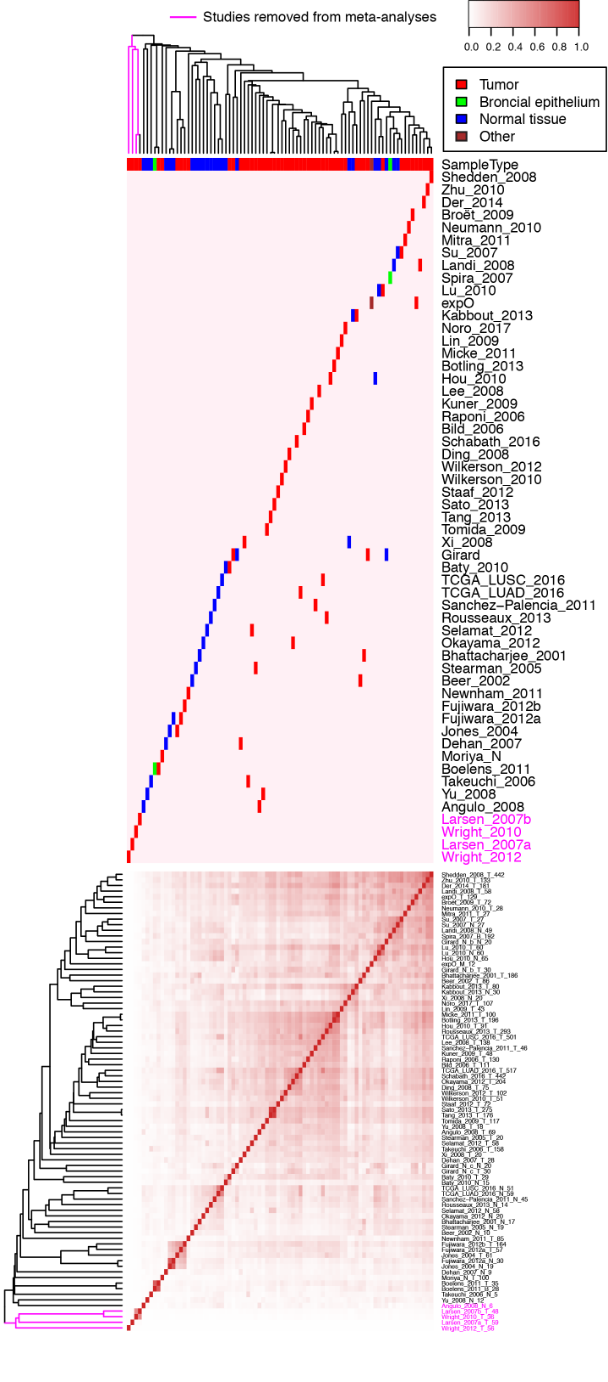


**FigS3. ICC Clustering of samples by sample type and sample source.**

82 tissue-and-study-specific expression datasets were generated from 56 studies and were used to perform quality control of expression datasets used for meta-analysis. Integrative correlation coefficients were calculated based on the global expression correlation among these 82 expression data sets and were visualized in the heatmap. Datasets were clustered by hierarchical clustering using the average linkage method. Column-side color labels of the heatmap highlight the location of datasets originating from specific studies, and different tissue types were represented by different colors. Four studies that had little ICC with all other studies were excluded from meta-analysis in LCE.

**References**

1. Irizarry RA, Bolstad BM, Collin F et al. Summaries of Affymetrix GeneChip probe level data. Nucleic Acids Res 2003; 31: e15.

2. Dai M, Wang P, Boyd AD et al. Evolving gene/transcript definitions significantly alter the interpretation of GeneChip data. Nucleic Acids Res 2005; 33: e175.

3. Liu G, Loraine AE, Shigeta R et al. NetAffx: Affymetrix probesets and annotations. Nucleic Acids Res 2003; 31: 82-86.

4. Arloth J, Bader DM, Roh S, Altmann A. Re-Annotator: Annotation Pipeline for Microarray Probe Sequences. PLoS One 2015; 10: e0139516.

5. Allen JD, Wang S, Chen M et al. Probe mapping across multiple microarray platforms. Brief Bioinform 2012; 13: 547-554.
