# Supplementary material for: LCE: an open web portal to explore gene expression and clinical associations in lung cancer
Source: Oncogene. 2018 Dec 7;38(14):2551–64. doi: 10.1038/s41388-018-0588-2 (PMC6477796; doi:10.1038/s41388-018-0588-2)
Supplement: Supplementary file 2 — Table S1 [file 41388_2018_588_MOESM2_ESM.pdf]

Table S1

Friendly_Name	PubmedID	GEO_ID	Sample_Number
Shedden_2008	18641660		442
Ding_2008	18948947	GSE12667	75
Tomida_2009	19414676	GSE13213	117
Zhu_2010	20823422	GSE14814	133
Lin_2009	19737969;20668451	GSE16534	43
Neumann_2010	20196851	GSE17475	28
Wilkerson_2010	20643781	GSE17710	51
Sanchez-Palencia_2011	20878980	GSE18842	91
Hou_2010	20421987	GSE19188	156
Lu_2010	20802022	GSE19804	120
Dehan_2007	17258348	GSE1987	37
Landi_2008	18297132	GSE10072	107
Fujiwara_2012b	21737174	GSE20853	164
Wright_2010	20544843	GSE20875	36
Fujiwara_2012a	21737174	GSE2088	87
expO		GSE2109	141
Wright_2012	22514692	GSE23822	56
Stearman_2005	16314486	GSE2514	39
Newnham_2011	21385341	GSE25326	85
Wilkerson_2012	22590557	GSE26939	102
Micke_2011	22011649	GSE28571	100
Staaf_2012	22676229	GSE29016	72
Kuner_2009	18486272	GSE10245	48
Rousseaux_2013	23698379	GSE30219	307
Okayama_2012	22080568;23028479	GSE31210	224
Bild_2006	16273092	GSE3141	111
Girard_N_b		GSE31547	50
Girard_N_c		GSE31548	50
Selamat_2012	22613842	GSE32863	116
Botling_2013	23032747	GSE37745	196
Spira_2007	17334370;20375364	GSE4115	192
Jones_2004	15016488;21737174	GSE1037	80
Sato_2013	3;24850841;27354471	GSE41271	275
Tang_2013	23357979	GSE42127	176
Kabbout_2013	23659968	GSE43458	110
Raponi_2006	16885343	GSE4573	130
Der_2014	24305008	GSE50081	181
Larsen_2007a	NA	GSE5828	59
Yu_2008	18636107	GSE5364	30
Larsen_2007b	17504995	GSE5843	48
Schabath_2016	26477306	GSE72094	442
Moriya_N		GSE7339	100
BroÃ«t_2009	19176396;20810387	GSE10445	72
Noro_2017	27613525	GSE74777	107
Su_2007	17540040	GSE7670	54
Angulo_2008	17992665	GSE8569	75
Lee_2008	19010856	GSE8894	138
Mitra_2011	21242119	GSE9971	27
Beer_2002	12118244		96
Bhattacharjee_2001	11707567		203
Baty_2010	19833826	GSE11117	44
TCGA_LUAD_2016	25079552		576
TCGA_LUSC_2016	22960745		552
Takeuchi_2006	16549822;21465578	GSE11969	163
Xi_2008	18927117	GSE12236	40
Boelens_2011	20832896	GSE12472	63
