# Supplementary material for: LCE: an open web portal to explore gene expression and clinical associations in lung cancer
Source: Oncogene. 2018 Dec 7;38(14):2551–64. doi: 10.1038/s41388-018-0588-2 (PMC6477796; doi:10.1038/s41388-018-0588-2)
Supplement: Supplementary file 5 — Table S4.1 [file 41388_2018_588_MOESM5_ESM.pdf]

Table S4.1

Variables in Patient table

1 Pat_ID — Primary ID

2

Pat_Gender	
Code	Gender
M	Male
F	Female
Null	unknown

3 Pat_Age — Integers

4

Pat_Race	
Code	Race
1	White
2	Black
3	Asian
4	Other
Null	Unknown

5

Pat_Smoking_Status	
Code	Smoking status
1	Smoker
2	Current Smoker
3	Former Smoker
4	Non-Smoker
Null	Unknown

6 Pat_Packs_Per_Year — Pack cigarettes smoked per year in Integers

7 Pat_Histology — Link to ID in Histology Codebook

8

Pat_Adjuvant_Chemo	
Code	Adjuvant chemotherapy treatment
1	Yes
0	No
Null	unknown

9

Pat_Neoadjuvant_Chemo	
Code	Neoadjuvant chemotherapy treatment
1	Yes
0	No
Null	unknown

10

Pat_Radiation	
Code	Radiation therapy treatment
1	Yes
0	No
Null	unknown

11 Pat_Overall_Survival_Months — Months of overall survival in integers

12

Pat_Died	
Code	Died
1	Yes
0	No
Null	unknown

13 Pat_Time_To_Recurrence — Months of recurrence-free survival in integers

14

Pat_Recurrence	
Code	Recurred
1	Yes
0	No
Null	unknown

15

Pat_T	
Code	TNM T stage
1	T1
2	T2
3	T3
4	T4
5	Tx
Null	unknown

16

Pat_N	
Code	TNM N stage
1	N0
2	N1
3	N2
4	Nx
Null	unknown

17

Pat_M	
Code	TNM M stage
1	M0
2	M1
3	Mx
Null	unknown

18

Pat_Stage	
Code	Tumor stage
I	I
I or II	I or II
II	II
III	III
IV	IV
Null	unknown

19

Pat_Substage	
Code	Tumor substage
A	A
B	B
Null	unknown

20

Pat_Grade	
Code	Tumor Grade
1	Well-differentiated
2	Moderately-differentiated
3	Poorly-differentiated
Null	unknown

21

Pat_EGFR_mutation	
Code	EGFR mutation
1	Yes
0	No
Null	unknown

22

Pat_KRAS_mutation	
Code	KRAS mutation
1	Yes
0	No
Null	unknown

23

Pat_ALK_translocation	
Code	ALK translocation
1	Yes
0	No
Null	unknown

24

Pat_TP53_mutation	
Code	TP53 mutation
1	Yes
0	No
Null	unknown

25

Pat_STK11_mutation	
Code	STK11 mutation
1	Yes
0	No
Null	unknown

26

Pat_Tumor_Site	
Code	Tumor anatomical site
MR	Right-Middle
UR	Right-Upper
LR	Right-Lower
UL	Left-Upper
LL	Left-Lower
Other	Other
Null	Unknwon
