# Supplementary material for: LCE: an open web portal to explore gene expression and clinical associations in lung cancer
Source: Oncogene. 2018 Dec 7;38(14):2551–64. doi: 10.1038/s41388-018-0588-2 (PMC6477796; doi:10.1038/s41388-018-0588-2)
Supplement: Supplementary file 6 — Table S4.2 [file 41388_2018_588_MOESM6_ESM.pdf]

Table S4.2

Variables in Sample table

1 Sam_Patient — Link to Pat_ID in Patient table

2 Sam_Platform — Link to Plat_ID in Platform codebook table

3

Sam_Experiment	
Code	Experiment Type
1	mRNA profiling

4 Sam_Dataset — Link to DS_ID in Dataset table

5

Sam_Normalization	
Code	Normalization Method
1	Re-annotated by RMA
2	reannotator-reannotated
3	Blast reannotated
4	author-processed

6

Sam_Normalization	
Code	Tissue type
1	Lung Tumor Tissue
2	Normal Lung Tissue
3	Bronchial Epithelium
4	Other

7 Sam_Name — Unique ID
