# Supplementary material for: LCE: an open web portal to explore gene expression and clinical associations in lung cancer
Source: Oncogene. 2018 Dec 7;38(14):2551–64. doi: 10.1038/s41388-018-0588-2 (PMC6477796; doi:10.1038/s41388-018-0588-2)
Supplement: Supplementary file 7 — Table S4.3 [file 41388_2018_588_MOESM7_ESM.pdf]

Table S4.3

Variables in mRNA_Expression table		
1	Gene_ID	Link to Entrez_ID in Gene_Info table
2	Sam_ID	Link to Sam_ID in Sample table
3	Expression	mRNA expression value in numerics
