# Supplementary material for: LCE: an open web portal to explore gene expression and clinical associations in lung cancer
Source: Oncogene. 2018 Dec 7;38(14):2551–64. doi: 10.1038/s41388-018-0588-2 (PMC6477796; doi:10.1038/s41388-018-0588-2)
Supplement: Supplementary file 8 — Table S4.4 [file 41388_2018_588_MOESM8_ESM.pdf]

Table S4.4

Variables in Dataset table		
1	DS_ID	Primary ID
2	DS_Accession	Accession ID from the original data source
3	DS_Overlaps	Sample overlap between datasets
4	DS_Gene_Number	Number of genes in expression data of this dataset
5	DS_Sample_Number	Number of samples in expression data of this dataset
6	DS_GEO_Title	Title in GEO database
7	DS_GEO_PubStatus	Date when GEO data became public
8	DS_GEO_SubmitDate	Date of GEO data submission
9	DS_LastUPdateDate	Date when the dataset was last updated from its source
10	DS_Plat_ID	Link to Plat_ID in Platform codebook table
11	DS_Pubmed_ID	Pubmed ID(s) of associated publication(s)
12	DS_Pubmed_Authors	Author(s) of associated pubilication(s)
13	DS_Pubmed_Title	Titles of associated publications(s)
14	DS_Pubmed_Journal	Journal name(s) of associated publication(s)
15	DS_PubDate	Date(s) of publication for associated publication(s)
16	DS_Friendly_Name	Friendly name used for dataset in LCE tools
17	DS_Has_Tumor_Tissue_Number	Number of tumor tissue samples
18	DS_Has_mRNA_Expression_Number	Number of genes with mRNA expression data available
19	DS_Has_Normal_Tissue_Number	Number of normal tissue samples
20	DS_Has_Gender_Number	Number of samples from patients with gender available
21	DS_Has_Age_Number	Number of samples from patients with age available
22	DS_Has_Race_Number	Number of samples from patients with race available
23	DS_Has_SS_Number	Number of samples from patients with smoking status available
24	DS_Has_PPY_Number	Number of samples from patients with number of cigarettes smoked pear year available
25	DS_Has_Histology_Number	Number of samples from patients with histology available
26	DS_Has_AC_Number	Number of samples from patients with adjuvant chemotherapy treatment information information available
27	DS_Has_NC_Number	Number of samples from patients with neoadjuvant chemotherapy treatment information available
28	DS_Has_Radiation_Number	Number of samples from patients with radiation chemotherapy treatment information available
29	DS_Has_OSM_Number	Number of samples from patients with overall survival in months available
30	DS_Has_Died_Number	Number of samples from patients with vital status available
31	DS_Has_TTR_Number	Number of samples from patients with recurrence-free survival in months available
32	DS_Has_Recurrence_Number	Number of samples from patients with recurrence status available
33	DS_Has_T_Number	Number of samples from patients with tumor T stage available
34	DS_Has_N_Number	Number of samples from patients with tumor N stage available
35	DS_Has_M_Number	Number of samples from patients with tumor M stage available
36	DS_Has_Stage_Number	Number of samples from patients with tumor stage available
37	DS_Has_Substage_Number	Number of samples from patients with tumor substage available
38	DS_Has_Grade_Number	Number of samples from patients with tumor grade available
39	DS_Has_EGFR_Mutation_Number	Number of samples from patients with EGFR mutation status available
40	DS_Has_KRAS_Mutation_Number	Number of samples from patients with KRAS mutation status available
41	DS_Has_ALK_Translocation_Number	Number of samples from patients with ALK translocation status available
42	DS_Has_Tumor_Site_Number	Number of samples from patients with tumor anatomical site available
43	DS_Has_TP53_Mutation_Number	Number of samples from patients with TP53 mutation status available
44	DS_Has_STK11_Mutation_Number	Number of samples from patients with STK11 mutation status available
45	DS_Comment	Comments
46	DS_Focus	Focus of the dataset (lung cancer specific or multi-cancer study)
47	CreateTime	Create time
48	UpdateTime	Last update time
