# Supplementary material for: LCE: an open web portal to explore gene expression and clinical associations in lung cancer
Source: Oncogene. 2018 Dec 7;38(14):2551–64. doi: 10.1038/s41388-018-0588-2 (PMC6477796; doi:10.1038/s41388-018-0588-2)
Supplement: Supplementary file 9 — Table S4.5 [file 41388_2018_588_MOESM9_ESM.pdf]

Table S4.5

Platform Codebook			
Plat_ID	GEO accession	Platform technology	Platform title
1	GPL96	in situ oligonucleotide	[HG-U133A] Affymetrix Human Genome U133A Array
2	GPL97	in situ oligonucleotide	[HG-U133B] Affymetrix Human Genome U133B Array
3	GPL570	in situ oligonucleotide	[HG-U133_Plus_2] Affymetrix Human Genome U133 Plus 2.0 Array
4	GPL91	in situ oligonucleotide	[HG_U95A] Affymetrix Human Genome U95A Array
5	GPL8300	in situ oligonucleotide	[HG_U95Av2] Affymetrix Human Genome U95 Version 2 Array
6	GPL17586	in situ oligonucleotide	[HTA-2_0] Affymetrix Human Transcriptome Array 2.0 [transcript (gene) version]
7	GPL5188	in situ oligonucleotide	[HuEx-1_0-st] Affymetrix Human Exon 1.0 ST Array [probe set (exon) version]
8	GPL6244	in situ oligonucleotide	[HuGene-1_0-st] Affymetrix Human Gene 1.0 ST Array [transcript (gene) version]
9	GPL1708	in situ oligonucleotide	Agilent-012391 Whole Human Genome Oligo Microarray G4112A (Feature Number version)
10	GPL6480	in situ oligonucleotide	Agilent-014850 Whole Human Genome Microarray 4x44K G4112F (Probe Name version)
11	GPL7015	in situ oligonucleotide	Agilent Homo sapiens 21.6K custom array
12	GPL9053	in situ oligonucleotide	Agilent-UNC-custom-4X44K
13	GPL8926	spotted oligonucleotide	PRHU05-S1-0006 (PC Human Operon v2 21k)
14	GPL15048	in situ oligonucleotide	Rosetta/Merck Human RSTA Custom Affymetrix 2.0 microarray [HuRSTA_2a520709.CDF]
15	GPL1293	spotted oligonucleotide	Hitachisoft AceGene Human Oligo Chip 30K Subset A
16	GPL6947	oligonucleotide beads	Illumina HumanHT-12 V3.0 expression beadchip
17	GPL6884	oligonucleotide beads	Illumina HumanWG-6 v3.0 expression beadchip
18	GPL962	spotted DNA/cDNA	CHUGAI 41K
19	GPL5645	spotted DNA/cDNA	CNIO Human Oncochip 2.0
20	GPL11205	spotted DNA/cDNA	PMCC Human 10.5K
21	GPL6650	spotted oligonucleotide	Novachip human 34.5k
22	GPL4723	spotted DNA/cDNA	SWEGENE_BAC_32K_Full
23	GPL80	in situ oligonucleotide	[Hu6800] Affymetrix Human Full Length HuGeneFL Array
24	GPL11154	high-throughput sequencing	Illumina HiSeq 2000 (Homo sapiens)
