# Supplementary material for: LCE: an open web portal to explore gene expression and clinical associations in lung cancer
Source: Oncogene. 2018 Dec 7;38(14):2551–64. doi: 10.1038/s41388-018-0588-2 (PMC6477796; doi:10.1038/s41388-018-0588-2)
Supplement: Supplementary file 10 — Table S4.6 [file 41388_2018_588_MOESM10_ESM.pdf]

Table S4.6

Histology Codebook				
ID	Level	Histology	parentID	parentHistology
10000000	0	Lung Tumors	Null	Null
11000000	1	Epithelial tumors	10000000	Null
11010000	2	Adenocarcinoma	11000000	Epithelial tumors
11010100	3	Lepidic adenocarcinoma	11010000	Adenocarcinoma
11010200	3	Acinar adenocarcinoma	11010000	Adenocarcinoma
11010300	3	Papillary adenocarcinoma	11010000	Adenocarcinoma
11010400	3	Micropapillary adenocarcinoma	11010000	Adenocarcinoma
11010500	3	Solid adenocarcinoma	11010000	Adenocarcinoma
11010600	3	Invasive mucinous adenocarcinoma	11010000	Adenocarcinoma
11010610	4	Mixed invasive mucinous and nonmucinous adenocarcinoma	11010600	Invasive mucinous adenocarcinoma
11010700	3	Colloid adenocarcinoma	11010000	Adenocarcinoma
11010800	3	Fetal adenocarcinoma	11010000	Adenocarcinoma
11010900	3	Enteric adenocarcinoma	11010000	Adenocarcinoma
11011000	3	Minimally invasive adenocarcinoma	11010000	Adenocarcinoma
11011010	4	Nonmucinous	11011000	Minimally invasive adenocarcinoma
11011020	4	Mucinous	11011000	Minimally invasive adenocarcinoma
11011100	3	Preinvasive lesions	11010000	Adenocarcinoma
11011110	4	Atypical adenomatous hyperplasia	11011100	Preinvasive lesions
11011120	4	Adenocarcinoma in situ	11011100	Preinvasive lesions
11011121	5	Nonmucinous	11011120	Adenocarcinoma in situ
11011122	5	Mucinous	11011120	Adenocarcinoma in situ
11020000	2	Squamous cell carcinoma	11000000	Epithelial tumors
11020100	3	Keratinizing squamous cell carcinoma	11020000	Squamous cell carcinoma
11020200	3	Nonkeratinizing squamous cell carcinoma	11020000	Squamous cell carcinoma
11020300	3	Basaloid squamous cell carcinoma	11020000	Squamous cell carcinoma
11020400	3	Preinvasive lesion	11020000	Squamous cell carcinoma
11020410	4	Squamous cell carcinoma in situ	11020400	Preinvasive lesion
12000000	1	Neuroendocrine tumors	10000000	Null
12010000	2	Small cell carcinoma	12000000	Neuroendocrine tumors
12010100	3	Combined small cell carcinoma	12010000	Small cell carcinoma
12020000	2	Large cell neuroendocrine carcinoma	12000000	Neuroendocrine tumors
12020100	3	Combined large cell neuroendocrine carcinoma	12020000	Large cell neuroendocrine carcinoma
12030000	2	Carcinoid tumors	12000000	Neuroendocrine tumors
12030100	3	Typical carcinoid tumor	12030000	Carcinoid tumors
12030200	3	Atypical carcinoid tumor	12030000	Carcinoid tumors
12040000	2	Preinvasive lesion	12000000	Neuroendocrine tumors
12040100	3	Diffuse idiopathic pulmonary neuroendocrine cell hyperplasia	12040000	Preinvasive lesion
12050000	2	Large cell carcinoma	12000000	Neuroendocrine tumors
12060000	2	Adenosquamous carcinoma	12000000	Neuroendocrine tumors
12070000	2	Sarcomatoid carcinomas	12000000	Neuroendocrine tumors
12070100	3	Pleomorphic carcinoma	12070000	Sarcomatoid carcinomas
12070200	3	Spindle cell carcinoma	12070000	Sarcomatoid carcinomas
12070300	3	Giant cell carcinoma	12070000	Sarcomatoid carcinomas
12070400	3	Carcinosarcoma	12070000	Sarcomatoid carcinomas
12070500	3	Pulmonary blastoma	12070000	Sarcomatoid carcinomas
12080000	2	Other and Unclassified carcinomas	12000000	Neuroendocrine tumors
12080100	3	Lymphoepithelioma-like carcinoma	12080000	Other and Unclassified carcinomas
12080200	3	NUT carcinoma	12080000	Other and Unclassified carcinomas
12090000	2	Salivary gland-type tumors	12000000	Neuroendocrine tumors
12090100	3	Mucoepidermoid carcinoma	12090000	Salivary gland-type tumors
12090200	3	Adenoid cystic carcinoma	12090000	Salivary gland-type tumors
12090300	3	Epithelial-myoepithelial carcinoma	12090000	Salivary gland-type tumors
12090400	3	Pleomorphic adenoma	12090000	Salivary gland-type tumors
12100000	2	Papillomas	12000000	Neuroendocrine tumors
12100100	3	Squamous cell papilloma	12100000	Papillomas
12100110	4	Exophytic	12100100	Squamous cell papilloma
12100120	4	Inverted	12100100	Squamous cell papilloma
12100200	3	Glandular papilloma	12100000	Papillomas
12100300	3	Mixed squamous and glandular papilloma	12100000	Papillomas
12110000	2	Adenomas	12000000	Neuroendocrine tumors
12110100	3	Sclerosing pneumocytoma	12110000	Adenomas
12110200	3	Alveolar adenoma	12110000	Adenomas
12110300	3	Papillary adenoma	12110000	Adenomas
12110400	3	Mucinous cystadenoma	12110000	Adenomas
12110500	3	Mucous gland adenoma	12110000	Adenomas
13000000	1	Mesenchymal tumors	10000000	Null
13010000	2	Pulmonary hamartoma	13000000	Mesenchymal tumors
13020000	2	Chondroma	13000000	Mesenchymal tumors
13030000	2	PEComatous tumors	13000000	Mesenchymal tumors
13030100	3	Lymphangioleiomyomatosis	13030000	PEComatous tumors
13030200	3	PEComa, benign	13030000	PEComatous tumors
13030210	4	Clear cell tumor	13030200	PEComa, benign
13030300	3	PEComa, malignant	13030000	PEComatous tumors
13040000	2	Congenital peribronchial myofibroblastic tumor	13000000	Mesenchymal tumors
13050000	2	Diffuse pulmonary lymphangiomatosis	13000000	Mesenchymal tumors
13060000	2	Inflammatory myofibroblastic tumor	13000000	Mesenchymal tumors
13070000	2	Epithelioid hemangioendothelioma	13000000	Mesenchymal tumors
13080000	2	Pleuropulmonary blastoma	13000000	Mesenchymal tumors
13090000	2	Synovial sarcoma	13000000	Mesenchymal tumors
13100000	2	Pulmonary artery intimal sarcoma	13000000	Mesenchymal tumors
13110000	2	Pulmonary myxoid sarcoma with EWSR1-CREB1 translocation	13000000	Mesenchymal tumors
13120000	2	Myoepithelial tumors	13000000	Mesenchymal tumors
13120100	3	Myoepithelioma	13110000	Myoepithelial tumors
13120200	3	Myoepithelial carcinoma	13110000	Myoepithelial tumors
14000000	1	Lymphohistiocytic tumors	10000000	Null
14010000	2	Extranodal marginal zone lymphomas of mucosa-associated lymphoid tissue (MALT lymphoma)	14000000	Lymphohistiocytic tumors
14020000	2	Diffuse large cell lymphoma	14000000	Lymphohistiocytic tumors
14030000	2	Lymphomatoid granulomatosis	14000000	Lymphohistiocytic tumors
14040000	2	Intravascular large B cell lymphoma	14000000	Lymphohistiocytic tumors
14050000	2	Pulmonary Langerhans cell histiocytosis	14000000	Lymphohistiocytic tumors
14060000	2	Erdheim-Chester disease	14000000	Lymphohistiocytic tumors
15000000	1	Tumors of ectopic origin	10000000	Null
15010000	2	Germ cell tumors	15000000	Tumors of ectopic origin
15010100	3	Teratoma, mature	15010000	Germ cell tumors
15010200	3	Teratoma, immature	15010000	Germ cell tumors
15020000	2	Intrapulmonary thymoma	15000000	Tumors of ectopic origin
15030000	2	Melanoma	15000000	Tumors of ectopic origin
15040000	2	Meningioma, NOS	15000000	Tumors of ectopic origin
16000000	1	Metastatic tumors	10000000	Null
20000000	0	Normal	Null	Null
30000000	0	Other	Null	Null
40000000	0	Unknown	Null	Null
