# Supplementary material for: LCE: an open web portal to explore gene expression and clinical associations in lung cancer
Source: Oncogene. 2018 Dec 7;38(14):2551–64. doi: 10.1038/s41388-018-0588-2 (PMC6477796; doi:10.1038/s41388-018-0588-2)
Supplement: Supplementary file 11 — Table S5 [file 41388_2018_588_MOESM11_ESM.pdf]

Table S5

	qPCR.measurement	qPCR.lower	qPCR.upper	Meta.analysis.model	Meta.analysis.lower	Meta.analysis.upper
NR2F2	-0.74	-1.28	-0.21	-1.30	-1.55	-1.06
NR2C2	-0.16	-0.67	0.36	-0.20	-0.60	0.20
NR0B1	0.46	-0.07	0.98	0.58	0.47	0.69
NR1H2	-0.27	-0.79	0.25	-0.69	-0.91	-0.47
RARA	-0.39	-0.91	0.14	-0.85	-1.20	-0.51
RXRB	0.28	-0.24	0.80	0.11	-0.05	0.28
NR1D1	0.51	-0.02	1.03	0.58	0.40	0.76
NR1D2	0.02	-0.50	0.54	-0.37	-0.60	-0.15
NR2F6	0.67	0.14	1.20	1.05	0.75	1.36
RORA	-0.35	-0.87	0.17	-0.89	-1.17	-0.61
NR3C1	-0.37	-0.89	0.15	-1.20	-1.48	-0.92
PPARG	-0.97	-1.52	-0.43	-1.73	-2.05	-1.40
NR2C1	-0.18	-0.70	0.34	-0.29	-0.49	-0.08
PPARD	-0.20	-0.72	0.32	0.47	0.28	0.65
RARG	0.25	-0.27	0.77	0.43	0.16	0.70
RORB	-0.27	-0.79	0.25	-0.22	-0.46	0.02
RXRA	-0.22	-0.74	0.29	-0.89	-1.10	-0.67
NR4A2	-0.69	-1.22	-0.15	-1.16	-1.39	-0.92
THRA	-0.67	-1.20	-0.14	-0.82	-1.14	-0.50
NR2F1	-0.94	-1.48	-0.39	-1.65	-2.06	-1.24
AR	-0.41	-0.93	0.12	-0.73	-0.98	-0.49
THRB	-0.27	-0.79	0.25	-0.61	-0.92	-0.29
ESRRA	0.42	-0.11	0.94	0.57	0.40	0.74
ESR1	0.26	-0.26	0.77	-0.02	-0.19	0.15
NR4A3	-0.97	-1.51	-0.42	-1.31	-1.60	-1.01
NR0B2	-1.35	-1.92	-0.78	-0.31	-0.65	0.03
NR1H3	0.18	-0.34	0.70	-0.25	-0.46	-0.04
NR3C2	-0.77	-1.31	-0.23	-1.33	-1.62	-1.04
PGR	-0.83	-1.37	-0.29	-0.98	-1.46	-0.50
VDR	0.85	0.31	1.39	0.90	0.60	1.21
RARB	-0.01	-0.53	0.50	-0.22	-0.48	0.03
NR6A1	0.21	-0.30	0.73	0.24	0.09	0.39
NR4A1	-0.76	-1.30	-0.23	-1.56	-1.80	-1.32
RORC	0.02	-0.50	0.53	0.07	-0.28	0.43
HNF4G	0.43	-0.09	0.95	0.86	0.58	1.13
PPARA	0.35	-0.17	0.87	0.50	0.36	0.63
RXRG	-1.19	-1.76	-0.63	-0.94	-1.35	-0.54
ESR2	0.35	-0.17	0.87	0.43	0.30	0.56
NR2E3	-0.38	-0.91	0.14	-0.13	-0.36	0.11
NR5A2	-0.67	-1.20	-0.14	-1.07	-1.37	-0.76
NR2E1	0.59	0.07	1.12	0.49	0.30	0.69
NR1I2	0.33	-0.19	0.85	0.24	0.11	0.38
NR5A1	0.43	-0.09	0.95	0.38	0.22	0.53
ESRRG	-0.05	-0.57	0.46	0.00	-0.19	0.20
ESRRB	-0.12	-0.64	0.40	0.15	0.00	0.31
NR1I3	0.88	0.34	1.42	0.21	0.00	0.41
NR1H4	-0.18	-0.70	0.33	-0.11	-0.40	0.18
HNF4A	0.34	-0.18	0.86	0.26	0.10	0.41
